# Advancing Neuroscience Education Scholarship: The Curriculum Resource Report

**DOI:** 10.1212/NE9.0000000000200277

**Published:** 2025-11-14

**Authors:** Nikita Chhabra, Jeremy J. Moeller, Roy E. Strowd

**Affiliations:** 1 Department of Neurology, University of Michigan, Ann Arbor;; 2 Department of Neurology, Yale University, New Haven, CT; and; 3 Department of Neurology, Wake Forest University School of Medicine, Winston-Salem, NC.

We are thrilled to announce the launch of *Neurology® Education*'s Curriculum Resource Report (CRR), a major new initiative led by J.J. Moeller and N. Chhabra to advance a new model of scholarly contribution in clinical neuroscience education. This initiative reflects the journal's commitment to recognizing and sharing high-quality, ready-to-implement curricula. It offers the educator community a growing repository to both disseminate their scholarly teaching work and discover adaptable resources that make teaching in neuroscience easier, more effective, and impactful.

The CRR was created to advance the mission of *Neurology® Education*—to enhance teaching and learning in clinical neuroscience education through dissemination of scholarship focusing on best practices and innovation.^1^ From its founding, the journal sought submission of manuscripts in the Curriculum Innovation category, with a focus on novel, outcome-based curriculum evaluations that demonstrate robust research outcomes.^2^ Over time, it became clear that there was a rich trove of educational materials that, though often smaller in scope and focused on foundational or common topics rather that innovations, nonetheless addressed critical needs and provide meaningful value to the community of neurology educators. Descriptions of the implementation of these resources reflect thoughtful application of educational theory to real-world teaching challenges. When carefully designed and critically evaluated, these resources provide immense value for educators seeking ready-to-implement tools. The CRR offers a peer-reviewed pathway for disseminating such work, highlighting usability while complementing existing research formats and maintaining standards of rigor and accessibility.

Neuroscience education scholarship is frequently limited by single-institution studies that remain siloed by learner level or local practice.^3^ These constraints limit collaboration across institutions and result in educators reinventing solutions in isolation, without the benefit of collective expertise. Existing outlets for peer-reviewed educational work are limited, particularly those specific to neuroscience education that provide fully developed, ready-to-implement materials. This further constrains dissemination. The CRR addresses this gap by offering a peer-reviewed format that prioritizes practical usability, thereby creating new opportunities for all clinical neuroscience educators to share their work in ways that meaningfully advance the field. For education scholarship, the method of dissemination matters: choosing the right venue, making materials accessible, and framing innovation in ways that others can adopt are as important as the content itself.^4^

The CRR reflects a vision for the future of neurologic education scholarship. When preparing a CRR, educators have an opportunity to critically interrogate their own work before dissemination: are objectives clearly aligned with outcomes? Are the chosen assessment measures the most appropriate for the specific educational goals and objectives? Could these resources add value in other learning and teaching contexts? Each submission is rigorously peer reviewed. This process is not intended to be the gatekeeper but to assure the quality, rigor, and credibility of disseminated curricula. Over time, the collective CRRs will form a living repository of educational practice, the Curriculum Repository, which avoids duplication and amplifies innovation. The CRR does more than publish teaching tools; it advances the broader discourse of our field, ensuring that curricular scholarship is both recognized and shared in ways that elevate clinical neuroscience education.
